# Malignant mesothelioma following repeated exposures to cosmetic talc: A case series of 75 patients

**DOI:** 10.1002/ajim.23106

**Published:** 2020-03-16

**Authors:** Theresa S. Emory, John C. Maddox, Richard L. Kradin

**Affiliations:** ^1^ Department of Pathology Peninsula Pathology Associates Newport News Virginia; ^2^ Department of Medicine (Pulmonary/Critical Care) Massachusetts General Hospital Boston Massachusetts; ^3^ Department of Pathology Massachusetts General Hospital Boston Massachusetts

**Keywords:** anthophyllite, females, mesothelioma, peritoneal, pleural, talc, tremolite

## Abstract

**Background:**

Asbestos is the primary known cause of malignant mesothelioma. Some cosmetic talc products have been shown to contain asbestos. Recently, repeated exposures to cosmetic talc have been implicated as a cause of mesothelioma.

**Methods:**

Seventy‐five individuals (64 females; 11 males) with malignant mesothelioma, whose only known exposure to asbestos was repeated exposures to cosmetic talcum powders, were reviewed in medical‐legal consultation. Out of the 75 cases, 11 were examined for asbestiform fibers.

**Results:**

All subjects had pathologically confirmed malignant mesothelioma. The mean age at diagnosis was 61 ± 17 years. The mean latency from exposure to diagnosis was 50 ± 13 years. The mean exposure duration was 33 ± 16 years. Four mesotheliomas (5%) occurred in individuals working as barbers/cosmetologists, or in a family member who swept the barber shop. Twelve (16%) occurred in individuals less than 45 years old (10 females; 2 males). Forty‐eight mesotheliomas were pleural (40 females; 8 males), 23 were peritoneal (21 females; 2 males). Two presented with concomitant pleural and peritoneal disease. There was one pericardial, and one testicular mesothelioma. The majority (51) were of the epithelioid histological subtype, followed by 13 biphasic, 8 sarcomatoid, 2 lymphohistiocytoid, and 1 poorly differentiated. Of the 11 individuals whose nontumorous tissues were analyzed for the presence of asbestiform fibers, all showed the presence of anthophyllite and/or tremolite asbestos.

**Conclusions:**

Mesotheliomas can develop following exposures to cosmetic talcum powders. These appear to be attributable to the presence of anthophyllite and tremolite contaminants in cosmetic talcum powder.

## 1 INTRODUCTION

Asbestos, a generic term for naturally occurring fibrous mineral silicates, is recognized as a carcinogen by the general medical and scientific communities. In 1960, Wagner et al^1^ reported a large series of malignant mesotheliomas in individuals who had been exposed to asbestos from a South African asbestos mine. It has been demonstrated that all types of asbestos and even brief and low‐dose exposures are capable of causing malignant mesothelioma.^2^, ^3^, ^4^ In the 1970s, several types of cosmetic talcum powder products were demonstrated to contain asbestos.^5^, ^6^, ^7^ Asbestos fibers in commercial talcum powder have also been shown to become airborne upon application, and repeated exposures to cosmetic talc were implicated as a cause of mesothelioma by Gordon et al.^8^ Recently, Moline et al,^9^ reported a series of 33 subjects with malignant mesothelioma, whose only known exposure to asbestos was cosmetic talc. We present 75 additional subjects, with malignant mesothelioma, whose only known exposure to asbestos was cosmetic talc.

## 2 METHODS

One hundred forty subjects with documented exposures to cosmetic talc were initially reviewed. Exposures were identified through sworn deposition testimonies and answers to sworn interrogatories provided from subjects, parents, and spouses. Sixty‐five subjects were excluded due to recalled occupational or paraoccupational exposures to other sources of asbestos. Seventy‐five subjects, whose only known exposure to asbestos was via cosmetic talc, were included for further examination. The asbestos content of talcum products and airborne asbestos concentrations during simulations of the usage of these products was determined in previously published studies.^10^, ^11^

Tissues from biopsies and/or debulking procedures were examined and the diagnosis of malignant mesothelioma was confirmed by a board‐certified pathologist (JCM, TSE, RLK). Immunohistochemical staining results for BAP‐1 were available in a few cases but was not routinely performed as a part of this study.

No efforts were made to reconstruct levels of exposure but all subjects had been repeatedly exposed over many years. Eleven cases were examined for the presence of asbestiform fibers (aspect ratio, ≥3:1) in sampled tissues. Nine subjects were examined both by analytical transmission electron microscopy (ATEM) and microprobe analysis (MA) (see Table 2), whereas two were examined by scanning electron microscopy (SEM) and MA (results not shown).

## 3 RESULTS

The pertinent data from the 75 subjects is shown in Table 1. All had pathologically confirmed malignant mesothelioma. Sixty‐four subjects were females, 11 were males. The mean age at diagnosis was 61 ± 17 years, with a range of 14 to 94 years. The mean exposure duration was 33 ± 16 years with a range of exposure from 6 to 65 years. The mean latency from time of first exposure to diagnosis was 50 ± 13 years with a range of 14 to 72 years. A total of 4 of the 75 cases (5%) occurred in barbers/cosmetologists, or in a family member who swept the barber shop. Twelve (16%) were 45 years old or younger (10 females, 2 males) at the time of diagnosis. Forty‐eight mesotheliomas were pleural (40 females; 8 in males); 23 peritoneal (21 females; 2 men). Two presented with both pleural and peritoneal disease. There was one pericardial (woman), and one testicular mesothelioma. The majority, 51 (68%) were of epithelioid subtype, 13 biphasic (17%), 8 sarcomatoid (11%), 2 lymphohistiocytoid (3%), and 1 poorly differentiated (1%). Treatment, therapeutic outcomes, and survival were not determined in this study.

**Table 1:** Seventy‐five mesothelioma cases exposed to talcum powder

Case	Sex	Year of diagnosis	Age at diagnosis	Mesothelioma site	Histology	Estimated years of use	Estimated years of latency
1	F	2017	72	Pleural	Epithelioid	20	57
2	F	2014	51	Peritoneal	Epithelioid	30	50
3	F	2017	50	Pleural	Lymphohistiocytoid	41	50
4	F	2017	57	Peritoneal	Epithelioid	30	52
5	F	2015	65	Pleural	Epithelioid	39	62
6	F	2017	39	Peritoneal	Sarcomatoid	15	39
7	F	2016	29	Pericardial	Epithelioid	29	29
8	F	2017	94	Pleural	Epithelioid	60	72
9	F	2015	80	Pleural	Epithelioid	19	59
10	F	2016	72	Pleural	Sarcomatoid	43	59
11	F	2013	66	Peritoneal	Epithelioid	20	52
12	F	2011	48	Pleural	Lymphohistiocytoid	13	21
13	F	2010	51	Peritoneal	Epithelioid	15	20
14	F	2018	55	Peritoneal	Epithelioid	40	42
15	M	2017	81	Pleural	Sarcomatoid	60	60
16	F	2018	56	Pleural	Epithelioid	48	52
17	F	2017	32	Peritoneal	Epithelioid	25	32
18	F	2017	89	Pleural	Sarcomatoid	40	42
19	F	2019	73	Peritoneal	Epithelioid	47	56
20	M	2016	70	Pleural	Poorly differentiated	50	55
21	F	2015	66	Pleural	Epithelioid	40	43
22	F	2016	45	Pleural	Epithelioid	10	45
23	F	2018	45	Peritoneal	Epithelioid	39	45
24	M	2015	67	Pleural + peritoneal	Epithelioid	35	60
25	M	2017	78	Peritoneal	Biphasic	50	62
26	F	2018	57	Peritoneal	Biphasic	25	57
27	F	2013	14	Peritoneal	Epithelioid	12	14
28	F	2016	67	Peritoneal	Epithelioid	15	59
29	F	2018	73	Pleural	Epithelioid	30	65
30	F	2018	76	Pleural	Biphasic	60	55
31	M	2017	39	Testis	Epithelioid	7	39
32	F	2018	57	Pleural	Sarcomatoid	57	57
33	F	2016	68	Pleural	Epithelioid	38	64
34	F	2017	80	Pleural	Epithelioid	50	60
35	F	2016	63	Pleural	Epithelioid	15	54
36	F	2017	58	Pleural	Biphasic	20	58
37	F	2017	71	Pleural	Biphasic	60	71
38	F	2014	70	Pleural	Epithelioid	41	39
39	F	2016	26	Peritoneal	Epithelioid	20	26
40	F	2016	35	Pleural	Epithelioid	35	35
41	F	2017	72	Pleural	Sarcomatoid	23	60
42	F	2016	68	Peritoneal	Epithelioid	65	68
43	F	2018	77	Pleural	Biphasic	30	55
44	M	2015	58	Plural	Biphasic	6	49
45	F	2017	72	Peritoneal	Biphasic	30	42
46	F	2017	59	Pleural + peritoneal	Epithelioid	15	44
47	F	2016	80	Pleural	Biphasic	16	52
48	M	2019	71	Pleural	Epithelioid	40	57
49	F	2017	72	Pleural	Biphasic	58	58
50	F	2017	43	Peritoneal	Epithelioid	43	43
51	F	2017	75	Peritoneal	Sarcomatoid	55	59
52	F	2015	30	Pleural	Epithelioid	20	20
53	F	2017	79	Pleural	Biphasic	65	61
54	F	2017	66	Peritoneal	Epithelioid	20	60
55	F	2015	64	Peritoneal	Epithelioid	40	40
56	F	2017	24	Pleural	Epithelioid	12	24
57	M	2017	72	Pleural	Epithelioid	30	56
58	M	2017	74	Peritoneal	Epithelioid	30	52
59	M	2015	30	Pleural	Epithelioid	20	30
60	F	2016	81	Pleural	Sarcomatoid	52	52
61	F	2017	58	Pleural	Epithelioid	58	58
62	F	2016	75	Pleural	Epithelioid	8	47
63	F	2011	88	Pleural	Epithelioid	21	71
64	F	2016	73	Peritoneal	Biphasic	41	60
65^a^	M	2017	64	Pleural	Epithelioid	18	40
66^a^	F	2014	69	Pleural	Epithelioid	16	60
67^a^	F	2014	44	Peritoneal	Epithelioid	30	39
68^a^	F	2016	68	Pleural	Epithelioid	53	52
69^a^	F	2016	72	Pleural	Epithelioid	40	51
70^a^	F	2016	67	Pleural	Epithelioid	37	53
71^a^	F	2017	58	Pleural	Epithelioid	41	46
72^a^	M	2016	44	Pleural	Epithelioid	43	44
73^a^	F	2017	51	Pleural	Epithelioid	28	49
74^a^	F	2015	47	Pleural	Epithelioid	15	40
75^a^	F	2014	62	Pleural	Biphasic	14	53

^a^ Tissue analysis performed.

For the 11 subjects whose tissues were examined by ATEM and ASEM, the analysis showed the presence of tremolite and/or anthophyllite in all 11 subjects (Table 2).

**Table 2:** Fiber detection in tissue digestion from nine cases of malignant mesothelioma

				Concentration (fibers per gram of wet tissue)	Limit of detection (fibers per gram of wet tissue)	Tissue digest weight (g)
Case	Mesothelioma site	Asbestos type	Tissues examined	Lung, lymph node, omentum, ovary	Lung, lymph node, omentum, ovary	Lung, lymph node, omentum, ovary
65	Pleural	Anthophyllite, tremolite	Lung, lymph node	8625	4313	0.08, 0.34
66	Pleural	Anthophyllite	Lung, lymph node	15 333, 23 000	7667, 1150	0.06, 0.06
67	Peritoneal	Anthophyllite, tremolite	Omentum, lymph node	1917, 1725	639, 1725	0.54, 0.20
68	Pleural	Anthophyllite, tremolite	Lymph node	3044	1015	0.82, 0.34
70	Pleural	Anthophyllite, amosite, chrysotile	Lymph node	17 250	3450	1.06
71	Pleural	Anthophyllite, tremolite	Lung, lymph node	4313, 857, 3451	2156, 857, 575	0.16
72	Pleural	Anthophyllite, tremolite	Lymph node	17 250	3450	0.02
74	Pleural	Anthophyllite, tremolite	Lung	2300	460	2
75	Pleural	Anthophyllite	Lung, ovary	3450, 2070	1150, 2070	0.6, 0.2

*Note*: All cases shown were examined by analytical transmission electron microscopy and structures analyzed by microprobe analysis.

## 4 DISCUSSION

The 75 individuals with malignant mesothelioma caused by asbestos in cosmetic talc is currently the largest series reported to date. Recently, Moline et al reported 33 cases of malignant mesothelioma attributed to exposures to cosmetic talc. Like Moline's work, most of mesotheliomas in the present series occurred in women. Several mesotheliomas occurred specifically in hairdressers/barbers. Similarly, the asbestos fiber types found by ATEM in the tissues examined were comparable to those found in laboratory testing for cosmetic talc.^10^, ^11^, ^12^

Mesothelioma is recognized as a “signal tumor” of asbestos exposure, that is, if a patient has mesothelioma, it should signal an inquiry into potential asbestos exposure. The presence of asbestos in talc deposits has been recognized since the late 1940s.^13^, ^14^ Since the 1960s, laboratory testing has identified asbestos in samples of cosmetic talc.^15^, ^16^ Studies have confirmed that the most common types of asbestos present in cosmetic talc are tremolite, anthophyllite, and chrysotile. Industrial asbestos products used in the United States generally contained chrysotile, amosite, and/or crocidolite,^17^ and anthophyllite and tremolite were rarely present.^18^

While the latency between exposure and diagnosis in the present study is similar to the average latency for the development of mesothelioma (50 years) reported in surveillance epidemiology and end results program (SEER) data,^19^ the average age at diagnosis in this report (61 years) is 11 years younger than that in the SEER data (72 years). In addition, fewer than 3% of mesotheliomas in the SEER data occurred in individuals less than 45 years of age, whereas 16% of mesotheliomas of the present study occurred in individuals less than 45 years of age, and 83% of these cases were in women.^20^

The present report of 75 cases, together with the 35 cases previously reported^8^, ^9^ currently brings the number of individuals with confirmed diagnoses of malignant mesothelioma following repeated exposure to cosmetic talcum powder to more than 100. The presence of anthophyllite and tremolite in the fiber analysis of tissues obtained from the 11 subjects in this series, is consistent with a source in cosmetic talc.

Unlike industrial or occupational exposure to asbestos, where materials have been regulated, exposure to asbestos in cosmetic talc has not been widely reported or recognized within the medical community or to the public. Cosmetic talc products are most frequently used by women in the United States, and while the incidence of mesothelioma in women is less than in men, the majority have previously been reported as “idiopathic,” indicating no recognized source of asbestos exposure. The present study supports the contention that asbestos exposure through the use of cosmetic talc accounts may account for an uncertain percentage of these cases.

The present study has several limitations. It is both retrospective and uncontrolled, and the cases were submitted in medico‐legal consultation, all of which potentially introduce bias. However, detailed deposition testimonies provide a level of detail concerning product exposure—including dates of exposure, duration, and frequency—that is rarely obtained in routine medical exposure histories, and which allowed for corroborating witness testimony in some cases. The strengths of the current series include its size, as malignant mesothelioma is a rare disease (1‐2 cases per 100 000), and its novelty, as exposures to cosmetic talc are rarely considered by most medical practitioners when they are eliciting an exposure history to asbestos.

The findings of the present and other recent studies suggest that cosmetic talc may be a cause of malignant mesothelioma. Large‐scale controlled studies will be required to assess the prospective risk of developing mesothelioma following repeated exposures to talc. Although cosmetic talcs are not currently regulated by the Food and Drug Administration, the poor prognosis of malignant mesothelioma may warrant regulation or the withdrawal of cosmetic talcs from the market, as nontoxic alternatives such as corn starch are presently available.

## CONFLICTS OF INTEREST

Drs Emory, Maddox, and Kradin have testified in asbestos litigation, primarily for plaintiffs.

## DISCLOSURE BY AJIM EDITOR OF RECORD

John D. Meyer declares that he has no conflict of interest in the review and publication decision regarding this article.

## AUTHOR CONTRIBUTIONS

JCM and RLK developed the concept and the design of the work. JCM initiated the acquisition and developed the initial data analysis. TSE reviewed the materials, performed the statistical analysis, and was the primary author of the manuscript. RLK revised and gave the final approval of the version to be published.

## ETHICS APPROVAL AND INFORMED CONSENT

As these cases were selected from medical‐legal consultation practice and no identifying information was included, there was no formal institutional consent nor informed consent required.
